# Authors’ response: low-voltage area ablation in atrial fibrillation

**DOI:** 10.1093/ehjopen/oeag113

**Published:** 2026-07-23

**Authors:** Akihiro Sunaga, Yohei Sotomi, Yasushi Sakata

**Affiliations:** Department of Cardiovascular Medicine, The University of Osaka Graduate School of Medicine, 2-2 Yamadaoka, Suita, Osaka 565-0871, Japan; Department of Cardiovascular Medicine, The University of Osaka Graduate School of Medicine, 2-2 Yamadaoka, Suita, Osaka 565-0871, Japan; Department of Cardiovascular Medicine, The University of Osaka Graduate School of Medicine, 2-2 Yamadaoka, Suita, Osaka 565-0871, Japan

**This correspondence refers to ‘Win ratio analysis of low-voltage area ablation in persistent atrial fibrillation: sub-analysis of SUPPRESS-AF’, by A. Sunaga *et al*., https://doi.org/10.1093/ehjopen/oeag024.**

**Authors’ response to the commentary of De Ponti *et al*., https://doi.org/10.1093/ehjopen/oeag112.**

We thank Dr. Roberto De Ponti and colleagues for their interest in our sub-analysis of the SUPPRESS-AF trial and for their thoughtful comments regarding left atrial low-voltage ablation (LVA) in patients undergoing first-time ablation for persistent atrial fibrillation (AF).

Our primary objective was to assess whether adjunctive LVA ablation provides incremental benefit beyond pulmonary vein isolation (PVI) in a real-world persistent AF population, and our results suggest that, under current procedural practices, such benefit is not readily apparent in the overall cohort.^1,2^ We agree that interpretation of our findings should consider the inherent limitations of win ratio analysis, including hierarchical outcome ordering and handling of ties, as well as the relatively small number of secondary endpoint events in our study. We also acknowledge that the exclusion of patients with very limited LVAs (<5 cm^2^) may have affected our ability to detect potential benefits in this subgroup.

Among all patients with left atrial low-voltage areas (LVAs) ≥5%, we were unable to demonstrate the efficacy of adjunctive LVA ablation. However, potential efficacy was observed in a sub-analysis with stringent patient selection based on left atrial diameter and LVA burden.^3,4^ As mentioned by Dr. Roberto De Ponti, even minimal extension of LVAs is associated with altered mechanical and electrical left atrial function. Indeed, AF patients with minimal LVAs (<5% of the left atrial surface) may already exhibit left atrial mechanical dysfunction associated with post-ablation recurrences.^5^ In contrast, the presence of LVAs has been reported to reflect a marker of global left atrial electrical degeneration^6^ and diffuse fibrosis,^7^ rather than an arrhythmogenic substrate. Accordingly, not all LVAs serve as direct arrhythmogenic substrates; some may act as markers of atrial cardiomyopathy. Patients with a large LVA burden or an enlarged left atrial diameter are more likely to contain LVAs that function as true arrhythmogenic substrates, which may help identify appropriate targets for ablation. Of course, this does not preclude the possibility that LVAs smaller than 5 cm^2^ may also contain arrhythmogenic tissue.

In our study, scar homogenization was achieved in only 78% of cases due to concerns regarding esophageal injury and potential damage to the Bachmann bundle. Additionally, atrial tachycardias (ATs) have been reported to arise from regions subjected to homogenization.^8^ Conversely, the ERASE-AF trial,^9^ which primarily employed isolation as the main strategy, demonstrated efficacy, indicating that isolation may be preferable to homogenization as an approach to LVAs, given the potential for homogenization to leave residual arrhythmogenic substrate. Pulsed field ablation, capable of producing more uniform and transmural lesions than radiofrequency,^10^ may offer advantages for the ablation of LVAs.

As emphasized by Dr. Roberto De Ponti, LVAs are closely associated with atrial cardiomyopathy and can serve as arrhythmogenic substrates, representing a potentially valuable target for ablation. Nevertheless, our subanalysis and prior studies suggest that routine adjunctive LVA ablation may not consistently provide clinical benefit. These observations highlight the importance of carefully defining LVAs, selecting appropriate patients, and standardizing ablation strategies in future studies to clarify the true value of LVA-targeted therapy.
